# The correlation between the frequent intake of dietary migraine triggers and increased clinical features of migraine (analytical cross-sectional study from Egypt)

**DOI:** 10.1038/s41598-024-54339-8

**Published:** 2024-02-20

**Authors:** Abdel-Ghaffar I. Fayed, Hossam Emam, Alyaa N. Abdel-Fattah, Reham M. Shamloul, Thanaa A. Elkholy, Ensaf M. Yassen, Eman Hamdy, Mohie-eldin T. Mohamed, Mahrous I. Seddeek, Elsayed Abed

**Affiliations:** 1 Department of Neurology, Faculty of Medicine, Al-Azhar University, Cairo, 11651 Egypt; 2 Department of Food Industries Technology, Faculty of Technology of Industry and Energy, Samannoud Technological University, Samannoud, 31621 Egypt; 3 Department of Nutrition and Food Science, Faculty of Home Economic, Al-Azhar University, Tanta, 31527 Egypt; 4 Department of Neurology, Faculty of Medicine, Cairo University, Cairo, 11559 Egypt; 5 Department of Neurology, Faculty of Medicine, Alexandria University, Alexandria, 21526 Egypt

**Keywords:** Migraine, Food habits, Disability, Dietary triggers, Neurology, Medical research, Neurological disorders, Nutrition disorders

## Abstract

Despite the high prevalence of primary headaches, the role of food in modifying clinical characteristics among migraine patients is often overlooked. The aim is to detect the correlation between adopting unhealthy dietary habits and migraine severity and identify foods that have a greater chance of triggering specific subtypes of migraine. The present study was a cross-sectional analytical study that was conducted at Kasralainy Hospital, Cairo University, headache clinic at Alexandria University Hospital, and Al-Azhar University Hospitals from January to June 2020. We included 124 patients fulfilling the ICHD-3 criteria for migraine. A full clinical profile for migraine headaches was reported using a headache sheet applied to the Al-Azhar University headache unit. A nutritionist obtained data collected about dietary habits using many reliable scales and questionnaires such as food frequently sheets questionnaire. Logistic regression and Pearson correlation coefficients have been used to identify foods that are more likely to be associated with increased clinical features of migraine. Our participants reported that the fried meat, fried chicken, processed meats, fava beans, falafel, aged cheese “Pottery salted cheese” and “Rummy cheese”, salted-full fatty cheese “Damietta cheese”, citrus fruits, tea, coffee, soft drinks, nuts, pickles, chocolate, canned foods, sauces, ice cream, smoked herring, in addition to the stored food in the refrigerator for many days were significantly associated with the diagnosis of chronic migraine CM compared to episodic migraine (EM). Margarine, pickles, and smoked herring were significantly associated with the diagnosis of migraine with aura (MA) compared to migraine without aura (MO). Adopting unhealthy eating habits was a more prevalent dietary consumption pattern among people with chronic migraines compared to those with episodic migraine.

## Introduction

Migraine is one of the oldest and most common global human experiences that have challenged medicine throughout the ages^1^, according to Stovner et al.^2^ the estimated global prevalence of migraine in the latest review of epidemiological headache studies is 14.0%. In Egypt, the estimated one-year prevalence of migraine is 17.3%” which nearly corresponds to the global prevalence rate^3^. Migraine is linked to gastrointestinal disorders, but the exact mechanisms behind this interaction remain unclear. Factors influencing this interaction include inflammatory mediators, gut microbiota, neuropeptides, serotonin pathway, stress hormones, and nutritional substances^4^. Sgro et al.^5^ refereed that there is potential for novel therapeutic targets for headache disorders if understanding of the GBI axis in their aetiology, pathogenesis and recovery is increased. Because there is a direct connection between the enteric nervous system and the central nervous system^6^, and some nutrients play an effective role in the modification of chronic pain associated with inflammation^7^; there’s a potential role of foods in triggering, preventing, and managing migraine.

We compared the dietary consumption patterns of four migraine subtypes and investigated whether increased daily consumption of certain foods “which are considered migraine triggers” differs according to migraine status and aura status. We focused on excessive consumption to see if increasing the daily intake of these foods may be associated with increased duration, intensity, and frequency of migraine attacks. The current study also investigates whether adopting bad eating habits leads to an increase in the degree of disability caused by migraine. The results of this study will lead to adequate measures to manage migraines nutritionally.

## Materials and methods

### Study design and participants

A cross-sectional analytical study was carried out among 124 subjects with migraines included in the study, according to Thompson^8^. Thompson equation^8^ for the calculation of sample size, 124 subjects with migraine were included in the study based on this formula:

$$n = \frac{{N \times P\left( {1 - P} \right)}}{{\left( {N - 1 \times \left( {d^{2} \div z^{2} } \right)} \right) + P\left( {1 - P} \right)}}$$

where; n = sample size, N = population size, Z = confidence level at 95% = (1.96), d = error proportion = (0.05), and p = probability (50 %) = (0.5).

With permission of the original authors; the English versions of questionnaires and scales were translated into Arabic by translation and back-translation, to lessen the individual's variability in applying assessment methods; each assessment method was done by the same investigator at both baseline and reassessment instance.

The subjects were recruited from headache clinics at Kasralainy Hospital, Cairo University, headache clinic at Alexandria University Hospital, and Al-Azhar University Hospitals from January to June 2020. We included 124 patients fulfilling the ICHD-3 criteria for migraine. A full clinical profile for migraine headaches was reported using a headache sheet applied to the Al-Azhar University headache unit. A nutritionist obtained data collected about dietary habits using many reliable scales and questionnaires.

### Inclusion criteria

- Migraine patients fulfill the International Headache Society (IHS) criteria for migraine^9^.
- Both sexes.
- Age above 18 years.

### Exclusion criteria

- Patients with memory problems or poor reporting.
- Patients with coexisting tension-type headaches, other headaches of the eye, ear, nose, and throat (ENT), and dental origin.
- Patients with other neurological diseases and/or clinically relevant disorders such as head injuries and epilepsy.
- Patients with medication overuse.
- Pregnant women.

### Study tools and our work

The patient's socioeconomic status was evaluated by the valid and reliable socioeconomic status scale for health research in Egypt^10^. The branching logic questionnaire for the automated classification of migraine^11^ was used to classify patients into migraine with aura (MA) and migraine without aura (MO) to standardize the diagnostic criteria of neurologists on the International Headache Society criteria, patients were asked about visual or physical symptoms, or both, which include blurring vision, seeing dark spot, zigzag lines, or blind light flashes, localized numbness around the mouth and in upper limbs, speech/language auras, and disability in one side of the body or hemiparesis.

The migraine disability assessment scale (MIDAS) was used to assess the severity of migraines and patients' disabilities which are caused by migraines^12^.

Data on migraine clinical features, frequency of attacks, duration of attacks, the age when migraine started, and the family history of migraine were collected from the migraine clinical examination sheet according to the hospital. Patients who have15 days or more of migraine per month are classified as having chronic migraines, while those who have less than 15 days per month are classified as having episodic migraines.

The food habits assessment scale^13^ was used to detect the correlation between migraine severities and adopting bad dietary habits. It consists of 14 questions about important food habits such as drinking water, skipping breakfast, and meal contents, and it has a respectable reliability in assessing eating habits.

One of the most famous and reliable retrospective methods of dietary assessment has been used, which is the food frequency questionnaire^14^. It measures the food intake from the past; it collects data on all foods and drinks that have already been consumed and their quantity, and it depends on the respondent’s memory so standard cups, spoons, and images of foods were used to help the respondent to remember and estimate how much food he ate. Local foods and foods considered migraine triggers have been targeted into the list, and citrus fruits were defined as lemons, oranges, tangerines, grapefruits, and tomatoes.

### Statistical analysis

Results were analyzed using the statistical package for social science (SPSS)^15^. Descriptive statistics such as; arithmetic mean, standard deviation, and range were used for quantitative variables, qualitative variables were expressed as percentages, and association measures available within cross tabs were used as tests of independence between the categorical variables, (chi-square) test was used for comparison among proportions, Independent T-test was used to compare between the two sample means with the assumption that variables are normally distributed, P < 0.05 was adopted as the level of significance.

Variables with significant results were included in multivariate logistic regression analysis and univariate analysis of the regression model; we calculated odds ratios and 95% confidence interval for high intake of each food item for subjects who experience migraine with aura compared to those who have migraine without aura and subjects who experience chronic migraine compared to those who have episodic migraine. We examined high intake to identify foods whose high consumption may have a higher chance of triggering specific types of migraine. Separate logistic regression models were constructed for each food item. Regarding the correlation between the score of the migraine disability assessment scale and the score of the food habits scale; the Pearson correlation coefficient was used.

- Pooled standard deviation: 5.522680508593631
- Effect size (Cohen’s d): − 0.9053574604251853

The negative sign of Cohen’s d indicates that the mean of group 1 is lower than the mean of group 2. With a Cohen’s d of approximately − 0.91, this suggests a large effect size according to common benchmarks. The post-hoc power of the study is approximately: 0.9988203118928556, with a value of approximately 0.999, this indicates that the study was indeed adequately powered to detect the reported minimum differences. A power value this high suggests that there was a very low risk of committing a Type II error (failing to reject the null hypothesis when it is false), and the study had a very high likelihood of detecting an effect if there was one present.

### Institutional review board statement: ethics approval and consent to participate

All procedures performed in the study were under the ethical standards of the institutional research committee and with the 1964 Helsinki Declaration and its later amendments. The approval of the Ethics Committee, Faculty of Medicine at Alexandria University “serial number: 0106126” was taken to implement the research under the ethical considerations of scientific research.

### Informed consent statement

Written informed consent was obtained from all participants involved in this investigation before conducting any study-related activities.

## Results

The current study recruited 124 migraine patients randomly to ensure a representative sample from those who met migraine diagnosis criteria. About 81.5% of participants were females, 18.5% males, migraine worsened in married housewives, ± 31 years, and with low educational and socioeconomic levels. About 62% of participants had (MA), 38% had (MO), 61% had (CM), and 39% had (EM). About 75% of daily headache clinic visitors were excluded, while the remaining 25% were included. Out of 124 participants, 72 were recruited from Al-Qasr Al-Aini Hospital in Cairo, 32 from Alexandria University Hospital, and 20 from Al-Azhar University Hospital. Despite low cultural and educational levels, no one who’s invited to participate in the study declined voluntarily to respond to our request. Table 1 points to statistically significant differences between all participants in age and gender according to the type of migraine, where, females suffered from migraine with aura (MA) and chronic migraine (CM) more than migraine without aura (MO) and episodic migraine (EM), also, ages of subjects with (MA) and (CM) were slightly younger than those who suffered from (MO) and (EM).

**Table 1:** The basic characteristics of participants according to migraine status.

Characteristics	Migraine without aura (n = 47)	Migraine with aura (n = 77)	P. value	Chronic migraine (n = 76)	Episodic migraine (n = 48)	P. value
Mean of age	36 years	31 years	0.043*	31 years	36 years	0.007*
Standard deviation	12 years	10 years		10 years	12 years	
Gender						
Female’s n (%)	32 (68.1%)	69 (89.6%)	0.003*	71 (93.4%)	30 (62.5%)	< 0.001*
Male’s n (%)	15 (31.9%)	8 (10.4%)		5 (6.6%)	18 (37.5%)	
Socioeconomic status						
Low n (%)	6 (12.8%)	21 (27.3%)		23 (30.3%)	4 (8.3%)	
Moderate n (%)	27 (57.5%)	41 (53.3%)	0.160	34 (44.8%)	34 (70.9%)	0.014*
High n (%)	14 (29.8%)	15 (19.5%)		19 (25.0%)	10 (20.8%)	
Family history of migraine n (%)	14 (29.8%)	29 (37.7%)	0.371	29 (38.2%)	14 (29.2%)	0.306
Duration of attacks						
Hours N (%)	23 (48.9%)	29 (37.7%)	0.391	4 (5.3%)	48 (100.0%)	< 0.001*
Days N (%)	21 (44.7%)	36 (46.8%)		57 (75.0%)	0 (0.0%)	
More than 7 days N (%)	3 (6.4%)	12 (15.6%)		15 (19.7%)	0 (0.0%)	
Frequency of attacks N (%)						
Daily	3 (6.4%)	12 (15.6%)	0.228	15 (19.7%)	0 (0.0%)	< 0.001*
Weekly	21 (44.7%)	36 (46.8%)		57 (75.0%	0 (0.0%)	
Monthly	23 (48.9%)	29 (37.7%)		4 (5.3%)	48 (100.0%)	
Migraine disability assessment scale N (%)						
Mild disability	18 (38.3%)	21 (27.3%)	0.351	5 (6.6%)	34 (70.8%)	< 0.001*
Moderate disability	7 (14.9%)	10 (13.0%)		3 (3.9%)	14 (29.2%)	
Severe disability	22 (46.8%)	46 (59.7%)		68 (89.5%)	0 (0.0%)	
Food habits scale N (%)						
Unhealthy dietary habits	16 (34.0%)	31 (40.3%)	0.799 0.946	47 (61.8%)	0 (0.0%)	< 0.001*
Moderate dietary habits	15 (31.9%)	23 (29.9%)		28 (36.8%)	10 (20.8%)	
Healthy dietary habits	16 (34.0%)	23 (29.9%)		1 (1.3%)	38 (79.2%)	
Mean of duration of illness	± 14.07 years	± 14.21 years		± 14.69 years	± 13.05 years	0.476
Mean of age when headache started	± 21.50 years	± 20.22 years	0.302	± 20.01 years	± 21.86 years	0.136
Number of meals/day						
0–2 meals/day N (%)	27 (57.4%)	55 (71.4%)	0.110	74 (97.4%)	8 (16.7%)	< 0.001*
≥ 3 meals/day N (%)	20 (42.6%)	22 (28.6%)		2 (2.6%)	40 (83.3%)	
Cups of drinking wter/day N (%)						
0–6 cups/day	22 (46.8%)	49 (63.6%)	0.066	65 (85.5%)	6 (12.5%)	< 0.001*
≥ 7 cups/day	25 (53.2%)	28 (36.4%)		11 (14.5%)	42 (87.5%)	
Eating breakfast every day	22 (46.8%)	32 (41.6%)	0.567	12 (15.8%)	42 (87.5%)	< 0.001*

*Statically significant as p < 0.05.

There were no statistically significant differences between all participants in the family history of migraine, duration of illness, and age when the headache started.

There were significant differences between (CM) and (EM) in socioeconomic status, duration of attacks, frequency of attacks, the score of migraine disability assessment scale, score of food habits scale, number of the consumed meals per day, cups of drinking water that they consumed daily, and the extent of their eagerness to eat breakfast every day; but these factors weren’t differing significantly between (MO) and (MA). Chronic migraines are significantly correlated to adopting bad eating habits, increasing the degree of severity and disability of migraines, increasing the duration and frequency of attacks, skipping meals specifically breakfast, and decreasing the daily intake of water. The score on the migraine disability assessment scale and the score on the food habits scale didn’t significantly differ between (MA) and (MO).

Table 2 point to statistically significant differences between subjects with (MA) and subjects with (MO) in their consumption rate of pickles, smoked herring, and margarine in favor of subjects with (MA), canned juices and non-citric fruits in favor of subjects with (MO), while, there were no statistically significant differences between them in their consumption rate of bread, rice, pasta, honey, eastern and western sweets, meat, chicken, fish, processed meat products, liver, eggs, beans, fried beans, lentils, oil, milk, yogurt, cheeses, bananas, citrus fruits, caffeine drinks, herbs, nuts, chocolate, canned foods, ice cream, aspartame, sauces, food stored in the fridge and fermented mullet. The beer, red wine, and pork consumption rate was zero in both groups.

**Table 2:** Food consumption patterns of participants according to the migraine aura status (n, %).

Food items	High Consumption (daily)		Moderate Consumption (weekly)		Low Consumption (monthly)		p-value
	Migraine without aura	Migraine with aura	Migraine without aura	Migraine with aura	Migraine without aura	Migraine with aura	
Starches group							
Brown bread	32 (68.1%)	55 (71.4%)	6 (12.8%)	9 (11.7%)	9 (19.2%)	13 (16.9%)	0.270
French bread	11 (23.4%)	16 (20.8%)	13 (27.6%)	25 (32.5%)	23 (48.9%)	36 (46.8%)	0.463
Rice/pasta	12 (25.5%)	22 (28.6%)	32 (68%)	54 (70.1%)	3 (6.4%)	1 (1.3%)	0.570
Desserts	2 (4.3%)	4 (5.2%)	8 (17%)	10 (13%)	37 (78.7%)	63 (81.9%)	0.169
Honey	3 (6.4%)	4 (5.2%)	3 (6.4%)	11 (14.3%)	41 (87.2%)	62 (80.5%)	0.131
Meat group							
Fried meat	1 (2.1%)	0 (0%)	7 (14.9%)	11 (14.3%)	39 (83%)	66 (85.7%)	0.178
Grilled meat	2 (4.3%)	2 (2.6%)	32 (68.1%)	47 (61.1%)	13 (27.7%)	28 (36.4%)	0.107
Grilled chicken	0 (0%)	1 (1.3%)	36 (76.6%)	50 (65%)	11 (33.4%)	26 (33.8%)	0.226
Fried chicken	1 (2.1%)	2 (2.6%)	10 (21.3%)	21 (27.3%)	36 (76.6%)	54 (70.1%)	0.683
Processed meats	2 (2.4%)	2 (2.6%)	5 (10.7%)	21 (27.3%)	40 (85.1%)	54 (70.1%)	0.078
Fried fish	0 (0%)	1 (1.3%)	7 (14.9%)	13 (16.9%)	40 (85.1%)	63 (81.9%)	0.090
Grilled fish	0 (0%)	1 (1.3%)	22 (46.8%)	21 (27.3%)	25 (53.2%)	55 (71.5%)	0.206
Liver	0 (0%)	0 (0%)	8 (17%)	15 (19.5%)	39 (82.9%)	62 (80.6%)	0.316
Boiled eggs	5 (10.6%)	14 (18.2%)	27 (57.4%)	39 (50.7%)	15 (31.9%)	24 (31.2%)	0.307
Fried eggs	3 (6.4%)	6 (7.8%)	21 (44.6%)	31 (40.3%)	23 (48.9%)	40 (52%)	0.613
Legumes group							
Fava beans or falafel	9 (19.1%)	18 (23.4%)	12 (25.5%)	23 (29.9%)	26 (55.3%)	36 (46.8%)	0.739
Lentils	0 (0%)	3 (3.9%)	21 (44.7%)	29 (37.7%)	26 (55.3%)	45 (58.5%)	0.237
Fat group							
Seed oil	47 (100%)	76 (98.7%)	0 (0%)	0 (0%)	0 (0%)	1 (1.3%)	0.433
Hydrogenated ghee	8 (17%)	25 (32.5%)	3 (6.4%)	5 (6.5%)	36 (76.6%)	47 (61.1%)	0.127
Dairy group							
Milk or yogurt	15 (31.9%)	21 (27.3%)	14 (29.8%)	19 (24.7%)	18 (38.3%)	37 (48.1%)	0.155
Skimmed cheese	2 (2.4%)	8 (10.4%)	13 (27.7%)	12 (15.6)	32 (68.2%)	57 (74%)	0.700
Full fatty salty cheese	11 (23.4%)	28 (36.4%)	10 (21.3%)	16 (20.8%)	26 (55%)	33 (42.9%)	0.271
Aged cheese	4 (8.5%)	7 (9.1%)	7 (14.9%)	13 (16.9%)	36 (76.6%)	57 (74%)	0.865
Vegetable group							
Fresh vegetables	35 (59.6%)	35 (45.5%)	15 (31.9%)	30 (39%)	4 (8.5%)	12 (15.6%)	0.587
Cooked vegetables	0 (0%)	4 (5.2%)	44 (93.6%)	62 (80.5%)	3 (6.4%)	11 (14.3%)	0.221
Fruit group							
Non citrus fruits	23 (48.9%)	26 (33.8%)	22 (46.8%)	41 (53.3%)	2 (4.3%)	10 (13%)	0.023*
Citrus fruits	12 (25.5%)	25 (32.5%)	13 (27.6%)	25 (32.5%)	22 (46.8%)	27 (35.1%)	0.469
Drinks group							
Tea	24 (51.1%)	36 (46.8%)	4 (8.6%)	9 (11.7%)	19 (40.4%)	32 (41.6%)	0.111
Coffee	8 (17%)	13 (16.9%)	4 (8.5%)	10 (13%)	35 (80.4%)	54 (70.1%)	0.253
Soft drinks	1 (2.1%)	7 (9.1%)	8 (17%)	11 (14.3%)	38 (80.8%)	59 (76.7%)	0.157
Canned juices	5 (10.6%)	7 (9.1%)	24 (51.1%)	26 (33.8%)	18 (38.4%)	44 (57.2%)	0.040*
Herbal drinks	8 (17%)	6 (7.8%)	7 (14.9%)	15 (19.5%)	32 (68.2%)	56 (72.8%)	0.082
Various foods							
Nuts	1 (2.1%)	1 (1.3%)	4 (8.5%)	12 (15.6%)	42 (89.4%)	64 (83.2%)	0.112
Pickles	6 (12.8)	21 (27.3%)	10 (21.3%)	22 (28.6%)	31 (66%)	34 (44.2%)	0.023*
Chocolate	2 (4.3%)	4 (5.2%)	14 (29.8%)	15 (19.5%)	31 (66%)	58 (75.4%)	0.552
Canned foods	4 (8.5%)	16 (20.8%)	7 (14.9%)	23 (29.9%)	36 (76.7%)	38 (49.4%)	0.123
Beer/pork/red win	0 (0%)	0 (0%)	0 (0%)	0 (0%)	0 (0%)	0 (0%)	–
Stored food in refrigerator	3 (6.4%)	2 (2.6%)	23 (48.9%)	33 (42.9%)	21 (44.8%)	42 (54.6%)	0.841
Sauces	1 (2.1%)	2 (2.6%)	6 (12.7%)	9 (11.7%)	40 (85.2%)	66 (85.7%)	0.141
Ice cream	1 (2.1%)	2 (2.6%)	11 (23.7%)	17 (22.1%)	35 (74.4%)	58 (75.4%)	0.287
Aspartame	1 (2.1%)	6 (7.8%)	0 (0%)	0 (0%)	46 (97.9%)	71 (92.2%)	0.298
Smoked herring	0 (0%)	0 (0%)	0 (0%)	7 (9.1%)	47 (99.9%)	70 (91%)	0.029*
Fermented mullet	0 (0%)	0 (0%)	0 (0%)	2 (2.6%)	47 (100%)	75 (97.4%)	0.635

*Statically significant as p < 0.05.

Table 3 point to statistically significant differences between subjects with (CM) and subjects with (EM) in their consumption rate of sweets, honey, fried meat, fried chicken, processed meats such as luncheon and sausage, grilled fish, beans, fried beans, hydrogenated ghee, aged cheese, salted-full fatty cheese, citrus fruits, tea, coffee, soft drinks, milk, yogurt, skimmed cheese, nuts, pickles, chocolate, canned foods, salad dressings, sauces, ice cream, smoked herring, fermented mullet and stored food in the refrigerator in favor of subjects with (CM), meaning that subjects with (CM) consumed these foods more than subjects with (EM). Also, there were statistically significant differences between subjects with (CM) and subjects with (EM) in their consumption rate of liver, cooked vegetables, boiled eggs, and fried eggs in favor of subjects with (EM), while, there were no statistically significant differences between them in their consumption rate of brown bread, white bread, rice, pasta, boiled or grilled chicken, boiled or grilled meats, fried fish, lentils, oil, fresh vegetables, non-citrus fruits, juices, herbal drinks and aspartame. The beer, red wine, and pork consumption rate was zero in both groups.

**Table 3:** Food consumption patterns of participants according to the frequency of attacks (n, %).

Food items	High consumption (daily)		Moderate consumption (weekly)		Low consumption (monthly)		p-value
	Chronic migraine	Episodic migraine	Chronic migraine	Episodic migraine	Chronic migraine	Episodic migraine	
Starches group							
Brown bread	57 (75%)	30 (62.5)	11 (14.5%)	4 (8.3%)	8 (10.5%)	14 (29.2%)	0.112
French bread	13 (17.1%)	14 (29.2%)	22 (29%)	16 (33.4%)	41 (54%)	18 (37.5%)	0.338
Rice/pasta	24 (31.6%)	10 (20.8)	50 (65.8%)	36 (75%)	2 (2.6%)	2 (4.2%)	0.462
Desserts	6 (7.9%)	0 (0%)	18 (23.7)	0 (0%)	52 (68.4%)	48 (99.9%)	< 0.001*
Honey	7 (9.2%)	0 (0%)	14 (18.4%)	0 (0%)	55 (72.3%)	48 (100%)	0.001*
Meat group							
Fried meat	1 (1.3%)	0 (0%)	18 (23.7%)	0 (0%)	57 (75%)	48 (100%)	0.001*
Grilled meat	2 (2.6%)	2 (4.2%)	51 (67.1%)	28 (58.4%)	23 (30.2%)	18 (37.5%)	0.400
Grilled chicken	1 (1.3%)	0 (0%)	52 (68.4%)	34 (70.8%)	23 (30.2%)	14 (29.2%)	0.597
Fried chicken	3 (3.9%)	0 (0%)	31 (40.8%)	0 (0%)	42 (55.2%)	48 (100%)	< 0.001*
Processed meats	4 (5.2%)	0 (0%)	26 (34.2%)	0 (0%)	46 (60.5%)	48 (100%)	< 0.001*
Fried fish	1 (1.3%)	0 (0%)	10 (13.1%)	10 (20.9%)	65 (85.6%)	38 (79.3%)	0.522
Grilled fish	1 (1.3%)	0 (0%)	31 (40.8%)	12 (25%)	44 (57.9%)	36 (75.1%)	0.010*
Liver	0 (0%)	0 (0%)	13 (17.1%)	10 (20.9%)	63 (82.9%)	38 (79.1%)	0.007*
Boiled eggs	11 (14.5%)	8 (16.7%)	40 (52.6%)	26 (54.1%)	25 (32.9%)	14 (29.3%)	0.037*
Fried eggs	5 (6.6%)	4 (8.3%)	32 (42.2%)	20 (41.7%)	39 (51.3%)	24 (50%)	0.029*
Legumes group							
Fava beans or falafel	25 (32.9%)	2 (4.2%)	33 (43.5%)	2 (4.2%)	18 (23.7%)	44 (91.6%)	< 0.001*
Lentils	1 (1.3%)	2 (4.2%)	28 (36.8%)	22 (45.8%)	47 (61.8%)	24 (50%)	0.195
Fat group							
Seed oil	75 (98.7%)	48 (100%)	0 (0%)	0 (0%)	1 (1.3%)	0 (0%)	0.425
Hydrogenated ghee	33 (43.4%)	0 (0%)	8 (10.5%)	0 (0%)	35 (46%)	48 (100%)	< 0.001*
Dairy group							
Milk or yogurt	26 (34.2%)	10 (20.9)	17 (22.4%)	16 (33.4%)	33 (43.4%)	22 (45.9%)	0.003*
Skimmed cheese	10 (13.2%)	0 (0%)	13 (17.1%)	12 (25%)	53 (69.8%)	36 (75.1%)	0.013*
Full fatty salty cheese	39 (51.3%)	0 (0%)	26 (34.2%)	0 (0%)	11 (14.5%)	48 (100%)	< 0.001*
Aged cheese	11 (14.4%)	0 (0%)	20 (26.3%)	0 (0%)	45 (59.1%)	48 (100%)	< 0.001*
Vegetable group							
Fresh vegetables	39 (51.3%)	24 (50%)	27 (35.5%)	18 (37.5%)	10 (13.1%)	6 (12.5%)	0.227
Cooked vegetables	0 (0%)	4 (8.3%)	64 (84.2%)	42 (87.5%)	12 (15.8%)	2 (4.2%)	0.015*
Fruit group							
Non citrus fruits	35 (46%)	14 (29.1%)	33 (43.4%)	30 (62.5%)	8 (10.5%)	4 (8.3%)	0.056
Citrus fruits	37 (48.7%)	0 (0%)	32 (42.1%)	6 (12.5%)	7 (9.2%)	42 (87.4%)	< 0.001*
Drinks group							
Tea	56 (73.6%)	4 (8.4%)	11 (14.5%)	2 (4.2%)	9 (11.8%)	42 (87.4%)	< 0.001*
Coffee	21 (27.7%)	0 (0%)	12 (15.8%)	2 (4.2%)	45 (56.6%)	48 (95.9%)	< 0.001*
Soft drinks	8 (10.5%)	0 (0%)	17 (22.4%)	2 (4.2%)	51 (58.1%)	46 (95.8%)	0.001*
Canned juices	6 (7.9%)	6 (12.5%)	28 (36.8%)	22 (45.8%)	42 (55.3%)	20 (41.6%)	0.185
Herbal drinks	10 (13.1%)	4 (8.3%)	18 (23.7%)	4 (8.4%)	48 (63.2%)	40 (83.4%)	0.116
Various foods							
Nuts	2 (2.6%)	0 (0%)	4 (5.3%)	12 (25%)	70 (92%)	38 (75%)	0.001*
Pickles	27 (35.5%)	0 (0%)	28 (36.8%)	4 (8.3%)	21 (27.6%)	44 (75%)	< 0.001*
Chocolate	6 (7.9%)	0 (0%)	29 (38.2%)	0 (0%)	41 (53.9%)	48 (100%)	< 0.001*
Canned foods	20 (26.3%)	0 (0%)	30 (39.5%)	0 (0%)	26 (34.2%)	48 (100%)	< 0.001*
Beer/pork/red win	0 (0%)	0 (0%)	0 (0%)	0 (0%)	0 (0%)	0 (0%)	–
Stored food in refrigerator	5 (6.5%)	0 (0%)	52 (68.4%)	4 (8.3%)	19 (25%)	44 (91.6%)	< 0.001*
Sauces	3 (3.9%)	0 (0%)	15 (19.7%)	0 (0%)	58 (76.3%)	48 (100%)	0.001*
Ice cream	3 (3.9%)	0 (0%)	28 (36.9%)	0 (0%)	45 (59.2%)	48 (100%)	< 0.001*
Aspartame	3 (3.9%)	4 (8.3%)	0 (0%)	0 (0%)	73 (96%)	44 (91.7%)	0.435
Smoked herring	0 (0%)	0 (0%)	7 (9.2%)	0 (0%)	69 (90.7%)	48 (100%)	< 0.001*
Fermented mullet	0 (0%)	0 (0%)	2 (2.6%)	0 (0%)	74 (97.4%)	48 (100%)	0.001*

*Statically significant as p < 0.05.

Table 4 points to the fact that food groups were included in the regression model; however, none of them were significant predictors for the type of migraine either chronic or episodic.

**Table 4:** Multivariate-adjusted* odds ratio of the high intake of food groups by the type of migraine (N = 124).

Food groups	B	SE	*Sig.	OR
Starches group	− 4.962	557.836	0.993	0.007
Legumes group	11.295	914.212	0.990	80.893
Fat group	0.796	186.559	0.997	2.217
Dairy group	− 93.078	43.641	0.983	0.000
Fruit group	7.950	16.284	0.996	28.874
Saturated fat and free sugar group	7.045	32.803	0.983	11.614

Tables 5, and 6 points to the multivariate-adjusted odds ratio of the high intake of each food item which was significantly associated with specific subtypes of migraine in univariate analysis for those who suffer from (CM) compared to those with (EM) and for those who suffer from (MA) compared to those with (MO); it had been found that fried meat, fried chicken, processed meats such as luncheon and sausage, fava beans, falafel, aged cheese, salted-full fatty cheese, citrus fruits, tea, coffee, soft drinks, nuts, pickles, chocolate, canned foods, sauces, ice cream, smoked herring, and stored food in the refrigerator for many days were significantly correlated with (CM) compared to (EM). Margarine, pickles, and smoked herring were significantly correlated with (MA) compared to (MO) and the canned juices were significantly correlated with (MO) compared to (MA).

**Table 5:** Multivariate-adjusted* odds ratio of the high intake of each food item for those who suffer from chronic migraine compared to those with episodic migraine (N = 124).

Food items	B	SE	Sig.	OR	95% CI	
					LL	UL
Deserts/sweets	0.354	0.229	0.123	1.425	0.909	2.234
Honey	0.495	0.361	0.171	1.641	0.808	3.331
Fried meats	0.725	0.357	0.042*	2.065	1.027	4.154
Fried chicken	1.288	0.458	0.005*	3.626	1.479	8.892
Processed meat products	1.168	0.415	0.005*	3.214	1.425	7.251
Grilled fish	0.410	0.266	0.124	1.507	0.894	2.538
Liver	0.162	0.225	0.470	1.176	0.757	1.826
Boiled egg	− 0.066	0.176	0.707	0.936	0.663	1.322
Fried egg	− 0.013	0.173	0.939	0.987	0.702	1.386
Fava beans & Falafel	0.787	0.265	0.003*	2.197	1.307	3.693
Hydrogenated ghee	1.165	0.598	0.051	3.207	0.993	10.354
Whole or skimmed milk	− 0.051	0.152	0.740	0.951	0.706	1.281
Yogurt	− 0.028	0.152	0.855	0.973	0.722	1.309
Skimmed cheese	− 0.015	0.187	0.935	0.985	0.683	1.420
Full Fatty Salty Cheese	1.105	0.345	0.001*	3.018	1.536	5.931
Aged cheese	0.744	0.284	0.009*	2.103	1.207	3.667
Cooked vegetables	− 0.468	0.311	0.133	0.626	0.340	1.153
Citrus fruits	2.210	0.802	0.006*	9.116	1.893	43.894
Tea	0.465	0.167	0.005*	1.592	1.147	2.209
Coffee	0.773	0.321	0.016*	2.165	1.155	4.060
Soft drinks	0.499	0.246	0.042*	1.647	1.017	2.668
Nuts & peanuts	− 0.664	0.264	0.012*	0.515	0.307	0.864
Pickles	1.100	0.364	0.003*	3.003	1.471	6.133
Chocolates	1.857	0.697	0.008*	6.407	1.636	25.094
Canned food	1.026	0.333	0.002*	2.789	1.451	5.359
Stored food in refrigerator	0.866	0.278	0.002*	2.376	1.377	4.100
Salad Sauces	0.973	0.415	0.019*	2.647	1.173	5.971
Ice cream & iced drinks	0.610	0.292	0.037*	1.841	1.039	3.262
Smoked herring	2.009	0.803	0.012*	7.459	1.546	35.987
Fermented mullet	1.562	0.878	0.075	4.767	0.853	26.630

Note: B, unstandardized coefficients; SE, standard error; OR, odds ratio; CI, confidence interval; LL, lower limit; UL, upper limit.

*Statistically significant at p ˂ 0.05.

**Table 6:** Multivariate-adjusted* odds ratio of the high intake of each food item for those who suffer from migraine with aura compared to those with migraine without aura (N = 124).

Food items	B	SE	Sig	OR	95% CI	
					LL	UL
Margarine	1.513	0.650	0.020*	4.540	1.270	16.233
Non-citric fruits	− 1.006	0.725	0.165	0.366	0.088	1.515
Canned juice	− 2.036	0.633	0.001*	0.131	0.038	0.451
Pickles	0.773	0.321	0.016*	2.165	1.155	4.060
Smoked herring	1.857	0.697	0.008*	6.407	1.636	25.094

Note: B, unstandardized coefficients; SE, standard error; OR, odds ratio; CI, confidence interval; LL, lower limit; UL, upper limit.

*Statistically significant at p ˂ 0.05.

Ice cream, soft drinks, and tea have more than 1.5 chance to correlate with (CM) compared with (EM), fried meat, fava beans, falafel, aged cheese, coffee, canned foods, sauces, and stored food in the refrigerator for many days have more than 2 chance to correlate with (CM) compared with (EM), fried chicken, processed meats, pickles, and salted-full fatty cheese have more than 3 chance to correlate with (CM) compared with (EM), chocolate has more than 6 chance, smoked herring has more than 7 chance, and the citrus fruits has more than 9 chance to correlate with (CM) compared with (EM). Pickles have more than 2 chances, margarine has more than 4 chances, and smoked herring has more than 6 chances to correlate with (MA) compared with (MO).

**Figure 1:**
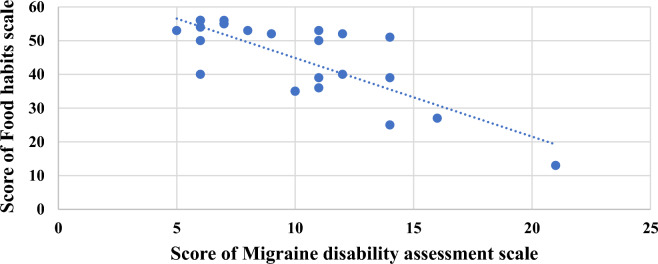
The correlation between migraine disability and food habits among patients with episodic migraine (N = 48).

**Figure 2:**
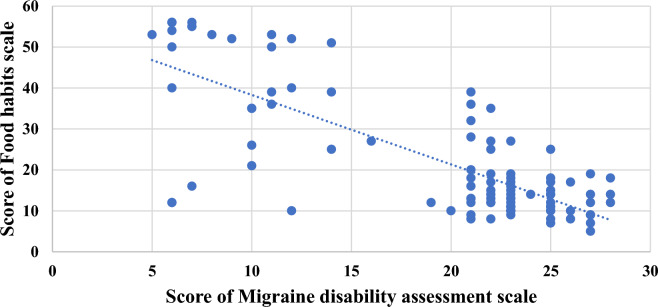
The correlation between migraine disability and food habits among all migraine patients (N = 124).

Table 7, Figs. 1 and 2 points to the correlation between the score on the food habits scale and the score on the migraine disability assessment scale; there was a statistically significant negative correlation between both scores among all participants “meaning that; healthier eating habits parallels with lower degree of severity and disability of migraine”. Also, among patients with episodic migraine, there was a statistically significant negative correlation between both scores. However, a non-significant negative correlation was present between both scores among patients with chronic migraine.

**Table 7:** The correlation between the score of the migraine disability assessment scale and the score of the food habits scale (N = 124).

Correlation	Chronic migraine (N = 76)	Episodic migraine (N = 48)	Total (N = 124)
Score of food habits scale	Scores on migraine disability assessment scale		
r: Pearson correlation	− 0.170	− 0.669	− 0.769
P: * Statistically significant	0.143	0.015*	0.001*

*Statically significant as p < 0.05.

## Discussion

Our results showed that fava beans and falafel “traditional Egyptian food consisting of beans and some vegetables are fried in trans saturated oil”, in addition to, ice cream, aged cheese, citrus, processed meats, and chocolates were the top dietary triggers of our participants, which nearly harmonize with Ozturan et al.^16^ who indicated that chocolate, aged cheese, coffee, tea, and alcohols are the main nutritional triggers of migraines, and Tai et al.^17^ who indicated that coffee, chocolate, and foods that rich in monosodium glutamate were main dietary triggers of migraine.

Foods that were significantly associated with chronic migraine were sweets, honey, fried meat, fried chicken, processed meats such as luncheon and sausage, grilled fish, fava beans, hydrogenated ghee, aged cheese, salted fatty cheese, citrus fruits, tea, coffee, soft drinks, pickles, chocolate, canned foods, sauces, ice cream, smoked herring, fermented mullet and the stored food in the refrigerator for days, which almost agrees with results of Nazari and Eghbali^18^.

Foods that were significantly associated with aura symptoms were pickles, smoked herring, and hydrogenated ghee, while Rist et al.^19^ found that chocolate, processed meats, wine, and dairy products were associated with the frequency of “migraine with aura”. Eating sweets and honey were associated with (CM) maybe because patients with migraines have an abnormal metabolism and abnormal transmission of glucose into brain cells, consequently a lack of mitochondrial energy in the brain and high levels of oxidative stress^20^.

Consumption of hydrogenated ghee, fried meat, and fried chicken was associated with (CM) because they contain omega-6 such as linoleic acid (LA), oleic acid (OA), and arachidonic acid (AA) which negatively affects migraines and reduces the amount of this free fatty acids in the diet has an effective role in preventing migraines^21^ because (AA) can stimulate *N*-methyl-d- aspartate receptors which stimulate migraines, omega-6 causes hyperpolarization and excessive sensitivity to physical pain due to the negative effects of omega-6 metabolites on the nervous system and immune system, in addition to omega-6 inhibits the anti-inflammatory effect of omega-3^22^, Increased serotonin release in the blood that occurs during migraines attacks is caused by increased the free fatty acids in the blood^23^.

Consumption of eggs was associated with (EM), and milk, yogurt, skimmed cheese, and nuts were associated with (CM), the reason may be due to allergic reactions to food antigens such as proteins of milk, grains, and nuts^24^ that activate the immune system then immune system attacks it with antibodies such as Immunoglobulin E (IGE), Immunoglobulin G (IGG) and Immunoglobulin M (IGM) that is cause meningitis, cerebral vasodilation, and consequently migraines^25^. Or because of food intolerance that causes migraines with a “brain and gut connection”^6^.

Current study showed that the excessive consumption of tea, coffee, chocolate and soft drinks were associated with increased severity, frequency and duration of migraines which is agree with Shimshoni^26^ who indicated that caffeine is a stimulant for neurons, but in a specific concentration of it causes neurotoxicity and cellular death of neurons, also, our results matched with Alstadhaug and Andreous^27^ who indicated that excessive and chronic consumption of caffeine causes increase in burden of migraine because it will causes brain hypersensitivity to migraine triggers due to the hypersensitivity of adenosine receptors, caffeine is antagonist of adenosine receptors and there are a great evidences that adenosine receptors involved in migraine occurrence, meaning that, it makes brain completely dependent on caffeine to reduce the activity of adenosine receptors, this condition is called “caffeine dependence syndrome”, which has been recognized by (WHO) as a behavioral disorder resulting from chronic and excessive consumption of more than 200 mg of caffeine per day, so, if the consumption of caffeine drinks suddenly stops or delays one day, the sudden withdrawal of caffeine will triggers migraines.

A current study showed that consumption of fava beans and falafel were associated with (CM), which may be due to that these foods contain a high concentration of biogenic amines such as histamine, putrescine, tyramine, and cadaverine^28^, pickles were associated with (MA) because it contains dangerous levels of tyramine and histamine that exceed the legal limits to the extent that causes health risks^29^. Aged cheese was associated with (CM) because 100 g of it contains 25 mg of tyramine^30^, tyramine and histamine are also found in soy sauce and fermented fish at a high concentration^31^ which explains why sauces and fermented mullets were associated with (CM). Citrus fruits such as oranges, kiwis, and grapefruit were associated with (CM), which may be due to these fruits containing a high concentration of biogenic amines such as histamine and putrescine reaching 200 mg/kg, particularly in bad storage conditions^28^, according to Pizza et al.^32^; biogenic amines are food antigens meaning that it is attacked by the immune system with antibodies which will cause a severe gastrointestinal disturbances which will triggering migraines by the mechanism of brain-gut connection. In addition, biogenic amines raise blood pressure^31^, have the same effect as serotonin and dopamine on migraines^33^ cause the release of adrenaline and norepinephrine which will cause hypertension, hyperglycemia, and migraine attacks^34^, and interact with migraine medications^30^ in one of the most important mechanisms that explain why these foods which contain biogenic amines were associated with increased the clinical features of migraine in our participants.

Chronic migraine was significantly observed in subjects who are used to eating stored food in a refrigerator for many days, it may be due to people with chronic migraine may feel worse and not feel like cooking, so the food sits instead of getting cooked; despite this, we would like to refer to a previous interpretation in which Gillman^30^, pointed out that biogenic amines such as histamine, tyramine, and phenyl ethyl amine ac- 307 cumulate in the stored food in the refrigerator due to the enzymatic break down of tyro- 308 sine by bacteria which are active in cooked food at a temperature above 5 °C and most household refrigerators cannot maintain a temperature below 5 °C which may triggering migraine. Gilman’s interpretation lacks sufficient empirical evidence to support the suggested association between chronic migraine and the consumption of stored food in refrigerators, and it doesn’t consider other potential factors that could contribute to this observation, such as lifestyle choices, access to fresh ingredients, or food preferences.

Increased consumption of smoked herring was associated with (CM); this may be due to that smoked herring contains carcinogenic compounds such as “Benzopyrene” which increases the oxidative stress of body cells^35^, Oxidative stress plays an important role in causing migraines because it causes damage in lymphocyte DNA, which will lead to an increase in the concentration of “urotensin receptors” in plasma which causes migraines by the cerebral vasoconstriction^36^.

Ice cream consumption has been associated with (CM), which is in line with the findings of Ferrara^37^, who observed a correlation between ice cream consumption and cold stimulus headaches. However, it is possible that the association between eating ice cream and (CM) could be due to allergic reactions to specific components present in its composition. This hypothesis is substantiated by the study conducted by Furlong et al.^38^, which reported that 14% of individuals who reported allergic reactions to peanut butter had visited an ice cream shop.

Our participants’ consumption of beer and red wine was zero, which contradicts Onderwater et al.^39^. According to Zaeem et al.^40^, alcohol contains high levels of histamine and it inhibits the enzyme that metabolizes histamine; accumulation of histamine in the blood triggers migraines, but our results may have shown the opposite due to religious considerations of the study community.

Processed meat and canned foods were significantly associated with increased clinical features of migraines, which is agreed with Doeun et al.^31^, this may be due to them containing monosodium glutamate (MSG) that causes gastrointestinal disturbances that trigger migraines^41^. Also, “glutamate” is an exciting neurotransmitter in the brain so an excessive level of it may become “neurotoxic” and lead to neuron death^42^ or because (MSG) raises blood pressure^43^ in addition to, the preservative substance “Nitrates” which is metabolized in the body to nitric oxide (NO), it’s well known that (NO) triggers migraines because it causes a cerebral vasodilation, spreading of the cortical depression and negatively participating in pain transfer in the central trigeminal pathway^40^.

Chocolate was significantly associated with (CM), which agrees with Lippi et al.^44^ who indicated that chocolate triggers and exacerbates migraines in 22.5% of patients. It may be due to a chocolate craving where people act upon, but not actually taking it which agrees with Magdalena et al.^45^ who found that Migraine patients often report chocolate as a trigger but there is insufficient evidence to support the claim that chocolate is a trigger for migraines.

The correlation between the migraine disability assessment scale and the food habits assessment scale showed that; whenever the dietary habits become worse, migraine severity and disability become more severe, which agrees with Bond et al.^46^ who found that modifying eating behaviors towards more healthy behaviors and practicing simple exercises led to reduce migraine frequency, and Costa et al.^47^ who found that weight loss and adopting a healthy diet significantly reduced migraine severity, and with Fila et al.^48^ who indicated that the genetic cause of migraines is aberrant DNA methylation, as vitamin B-9 (folic acid) can be adjust the aberrant DNA methylation, so migraine can be treated with “folate- rich, DNA methylation- directed diet”.

In our study, it was observed that none of the participants consumed alcohol, which aligns with the findings of Błaszczyk et al.^49^, who reported an inverse association between alcohol consumption and migraines. This suggests that individuals with migraines tend to avoid alcohol, and alcohol does not play a protective role against migraines^49^. In conclusion, Analytical cross-sectional studies are recognized good practices to measure the association between an exposure and a disease, condition, or outcome within a defined population according to Higgins et al.^50^ However, further research is needed to validate and expand upon our findings in order to enhance our understanding of this association.

## Conclusions

Adopting unhealthy eating habits was a more prevalent dietary consumption pattern among people with chronic migraines compared to those with episodic migraine. Fried meat, fried chicken, processed meats, fava beans, falafel, aged cheese, salted-full fatty cheese, citrus fruits, tea, coffee, soft drinks, nuts, pickles, chocolate, canned foods, sauces, ice cream, smoked herring, and stored food in the refrigerator for many days were significantly associated with (CM) compared to (EM). Margarine, pickles, and smoked herring were significantly associated with (MA) compared to (MO).

## Recommendations

Our study has limitations due to its cross-sectional design, which only allows for associations and not causality. Self-reporting and individual variations in migraine triggers may introduce bias. Confounding factors and unmeasured variables were not accounted for. Caution is needed in interpreting the findings, and further research is required to understand the complex relationship between diet and migraines, additionally, it’s important to note that the notion of an elimination diet is discouraged in migraine management, as it may lead to unnecessary hypervigilance. Instead, a wholesome meal that is regularly timed is preferred for controlling migraine.

## Limitations

One limitation of this study is that we did not include an explicit discussion of the model fitness assumptions and tests in the manuscript; additionally, we did not address the need for correcting multiple testing. This omission was primarily due to space limitations and our focus on presenting the core findings. However, it is important to note that assessing and validating these assumptions play a crucial role in the accuracy and reliability of logistic regression models. Future research should consider incorporating a comprehensive analysis of the model fitness assumptions and conducting appropriate tests to ensure the robustness of the findings. Additionally, as the study design is cross-sectional, it does not allow for the examination of the temporal relationship between food intake and migraine burden.

## Data Availability

All datasets generated and analyzed during the current study are not publicly available, but are available by reasonable request from the corresponding author.
